# Supplementary material for: Quantitative essentiality in a reduced genome: a functional, regulatory and structural fitness map
Source: Mol Syst Biol. 2025 Aug 13;21(10):1388–416. doi: 10.1038/s44320-025-00133-1 (PMC12494982; doi:10.1038/s44320-025-00133-1)
Supplement: Supplementary file 7 — Appendix [file 44320_2025_133_MOESM7_ESM.pdf]

Appendix for "Quantitative essentiality in a reduced genome: a functional, regulatory and structural fitness map"

<u>Table of contents:</u>

Appendix figures 2

Appendix Figure S1 2
Appendix Figure S2 3
Appendix Figure S3 4
Appendix Figure S4 5
Appendix Figure S5 6
Appendix Figure S6 7
Appendix Figure S7 8
Appendix Figure S8 9
Appendix Figure S9 10
Appendix Figure S10 11
Appendix Figure S11 12
Appendix Figure S12 13
Appendix Figure S13 15
Appendix Figure S14 17
Appendix Figure S15 18

Appendix figures

Appendix Figure S1

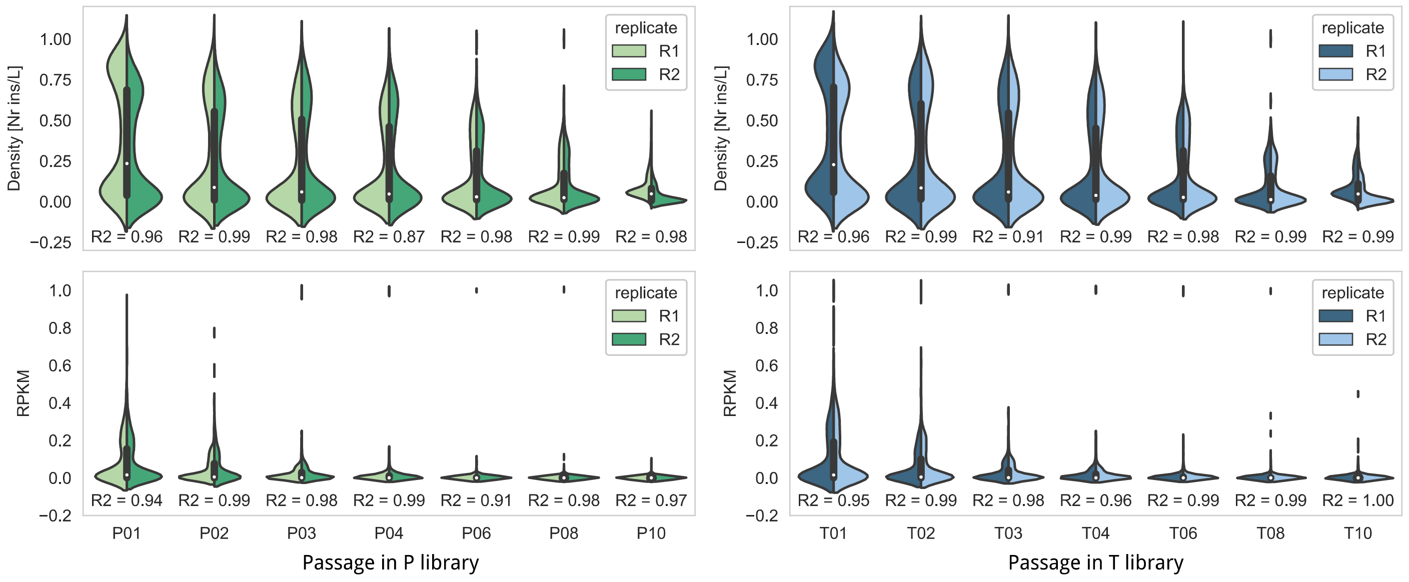

Appendix Figure S1 - Density and RPKM reproducibility between replicates. Comparative violin plot between replicates of the P (left) and the T libraries (right) at gene level. The top row represents the linear densities (total insertions normalized by gene length) across passages, while RPKM is represented at the bottom row. X-axes represent the increasing number of passages for each library condition.

Appendix Figure S2

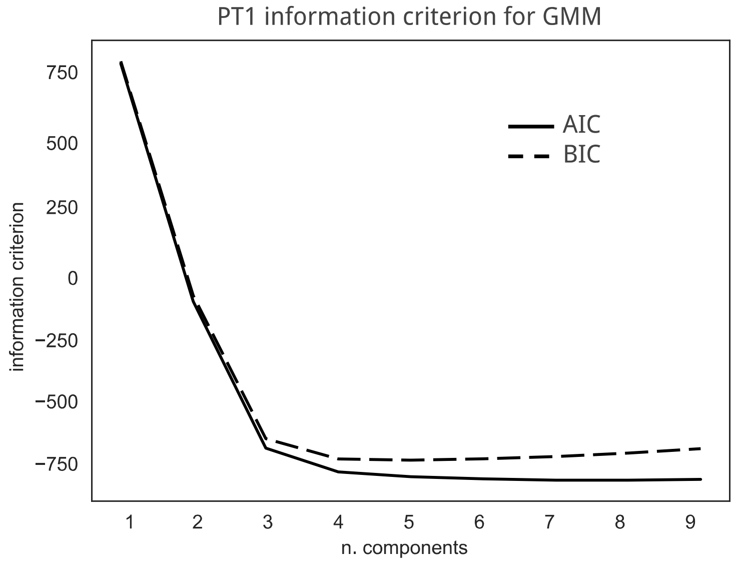

Appendix Figure S2 - AIC and BIC for GMM of 4 components in PT1. For each number of components tested (X-axis) the information criterion (IC; Y-axis) as Akaike Information criterion (AIC) and Bayesian Information Criterion (BIC) is measured. Ideally, a good classification needs as many components as required to minimize these values and reach the plateau to avoid overfitting.

Appendix Figure S3

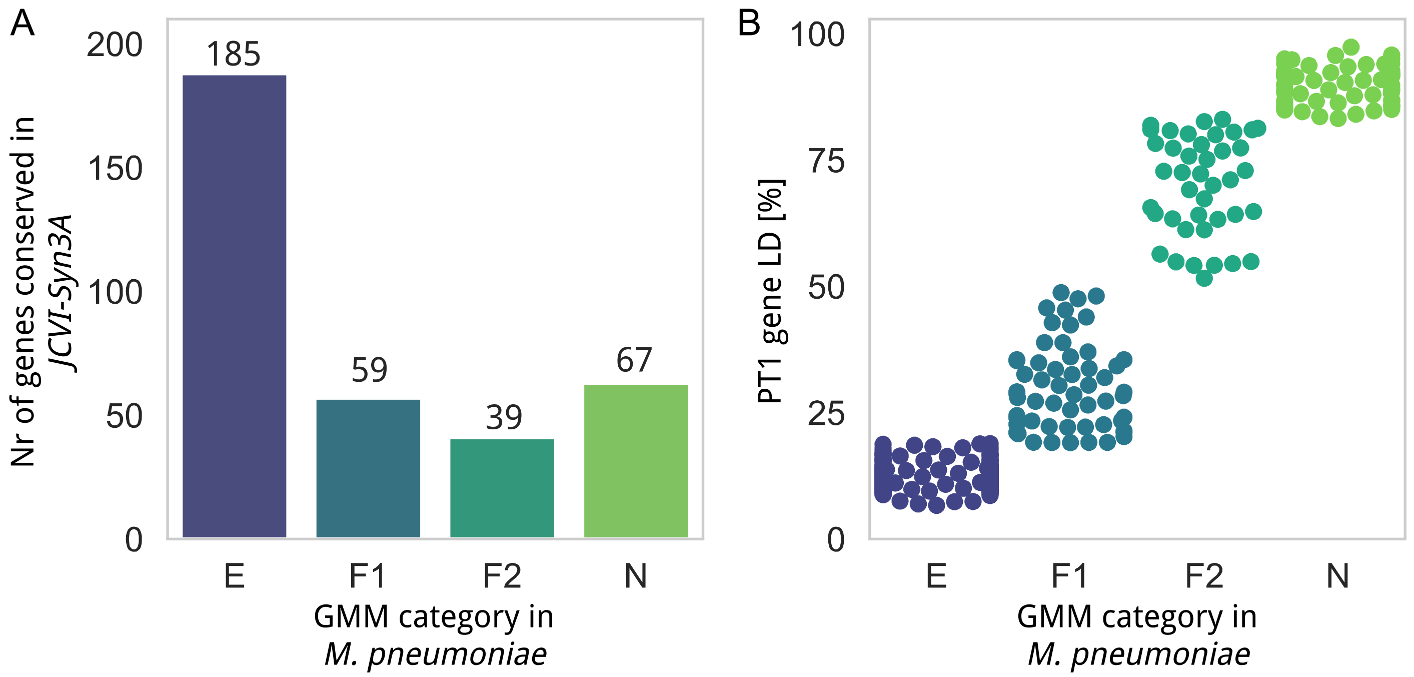

Appendix Figure S3 - Essentiality analysis of *M. pneumoniae* protein coding genes conserved in the Synthetic *M. mycoides* JCVI-Syn3A strain. A) Number of genes conserved in *M. pneumoniae and M. mycoides* JCVI-Syn3A (Y-axis) by BlastP homology, and represented by their essentiality category in *M. pneumoniae* at PT1 condition (X-axis). **B)** Swarm plot where each dot represents the linear density at PT1 (Y-axis) of a conserved gene colored by essentiality categories (X-axis).

Appendix Figure S4

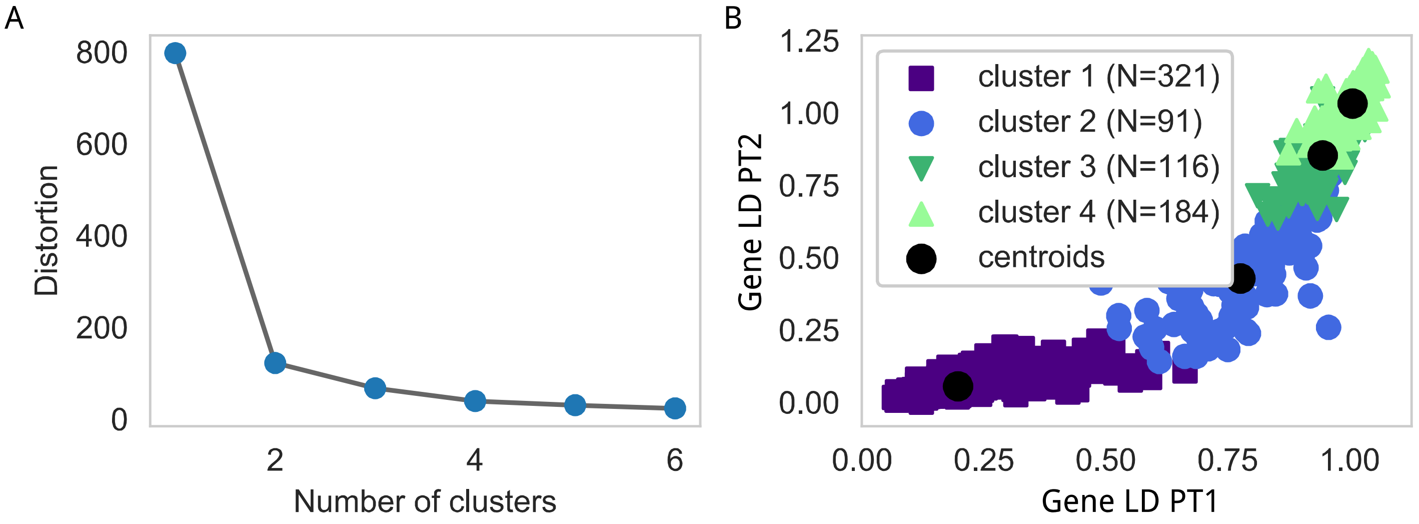

Appendix Figure S4 - K-means decay clustering for protein coding genes using dataset PT1 to PT8. A) Change in the distortion (Y-axis; it measures the difference between elements within proposed clusters) by testing the model from 1 to 6 clusters (X-axis). **B)** Scatter plot relating the LDs measured at passage 1 (X-axis) and passage 2 (Y-axis) colored by the cluster assigned by k-means, from cluster 1 (purple; genes that do not maintain insertions) to 4 (light green; genes that maintain insertions). Legend shows the cluster name, color assigned, and total number of genes grouped in each cluster. Note that clusters 1 and 2 are mainly different due to the persistence of insertions after the first passage.

Appendix Figure S5

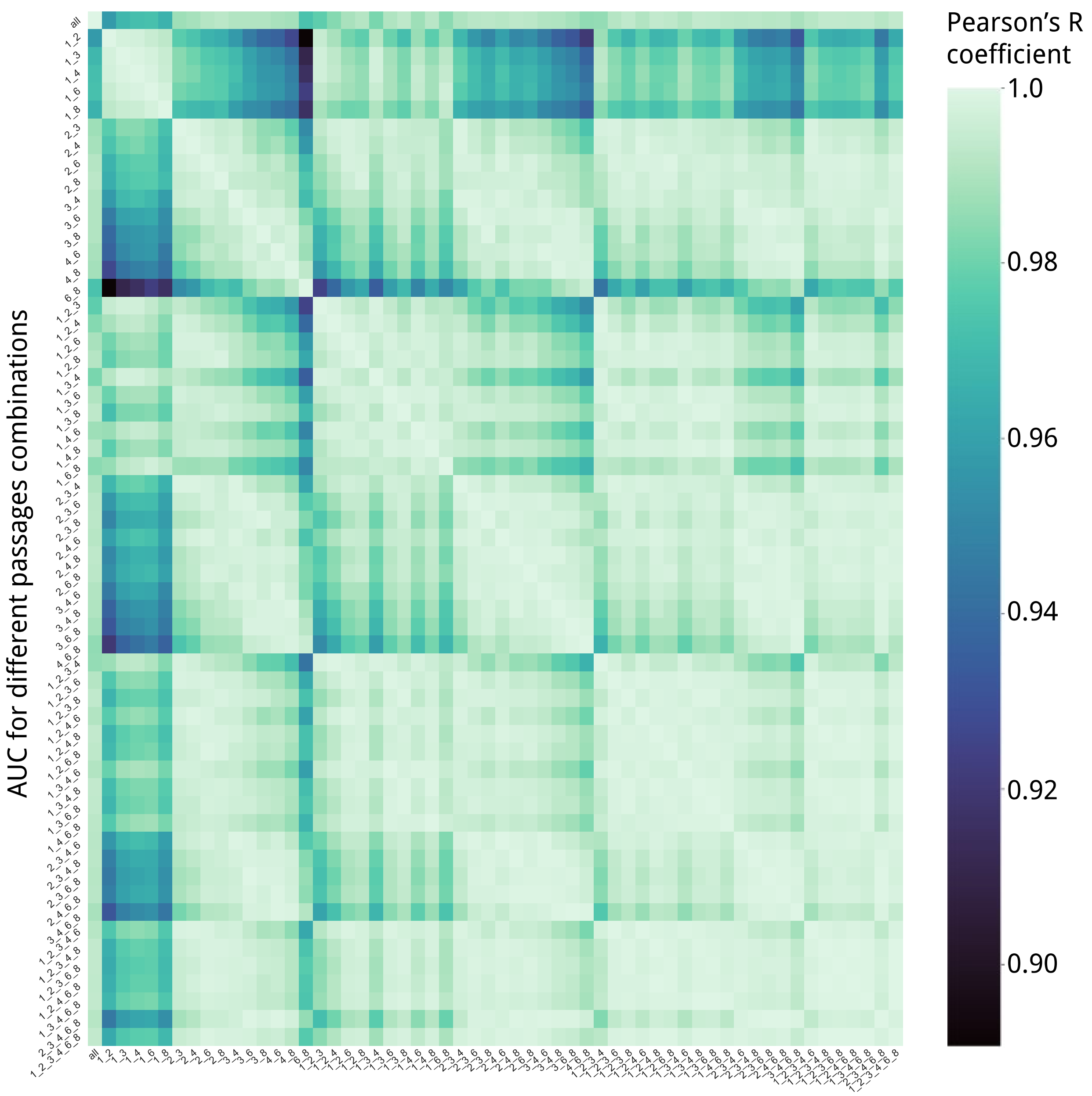

Appendix Figure S5 - AUC correlations with different passages. Heatmap representing Pearson's standard correlation coefficients between AUC calculations made by subsetting different combinations of PT passage profiles, from pairs (*e.g.*, 1_2 means AUC is calculated only between passages 1 and 2) to all ('all'). Color is lighter for higher correlation values which are always above 0.85 for every comparison.

Appendix Figure S6

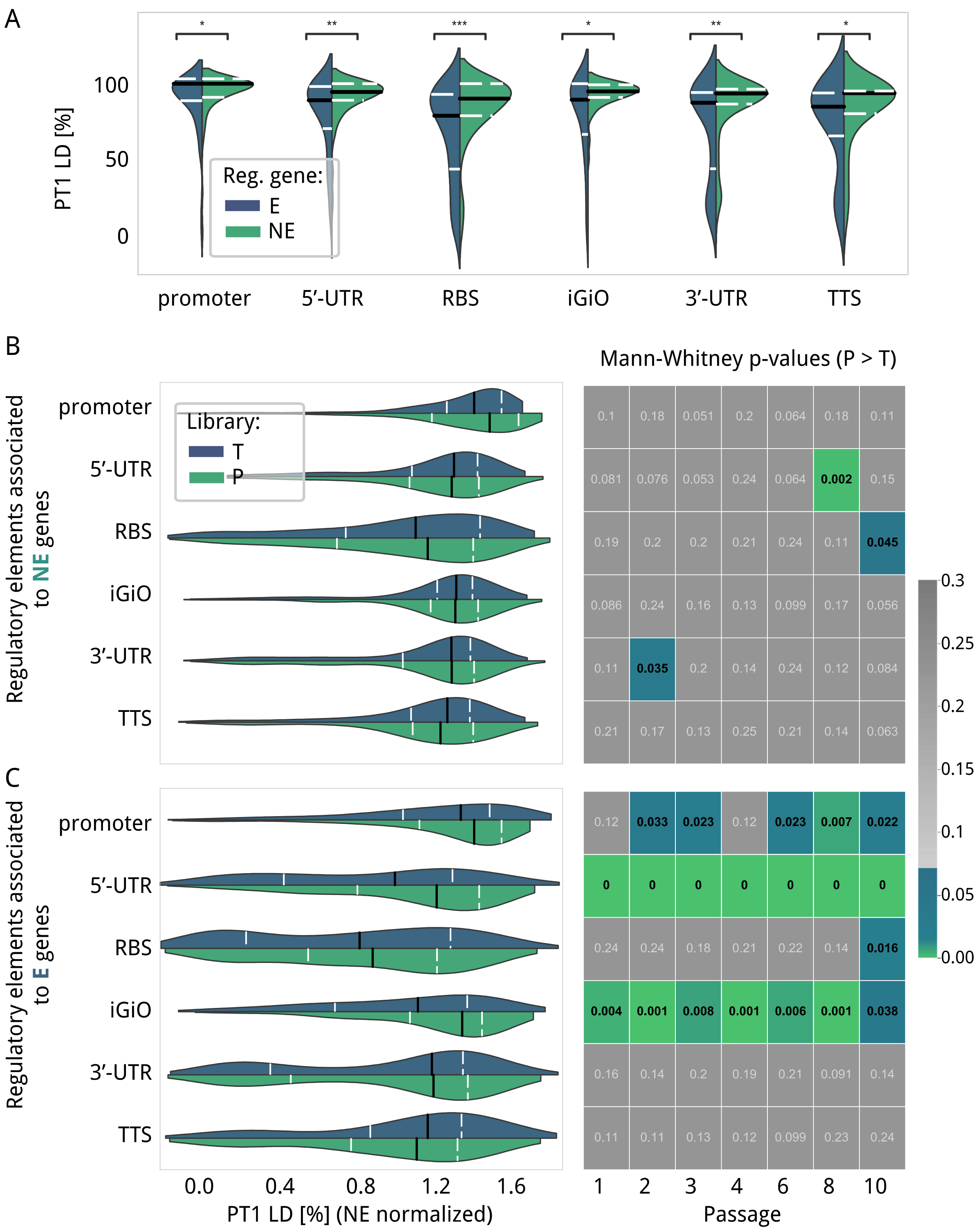

Appendix Figure S6 - Exploration of regulatory regions by linear density (LD). A) Comparative violin plot of LD measured in PT1 library for different regulatory elements (X-axis) associated with E/F1 or F2/NE regulated genes (blue and green, respectively). **B)** and **C)** Linear density comparison between P and T libraries for regulatory elements associated with NE (panel B) or E (panel C) genes. Left violin plots show the distribution of LD (X-axis) at P1 (green) and T1 (blue). Within the violins, white dashed lines represent the 1st and 3rd quartiles while the median is represented in black. Values are normalized by the LD found in NE regions. On the right, heatmaps with the *P* values from one-tailed Mann-Whitney tests are provided for each passage (X-axis) comparing P and T libraries. If significant by Mann-Whitney one-tailed test, the cell is colored while they remain gray otherwise.

Appendix Figure S7

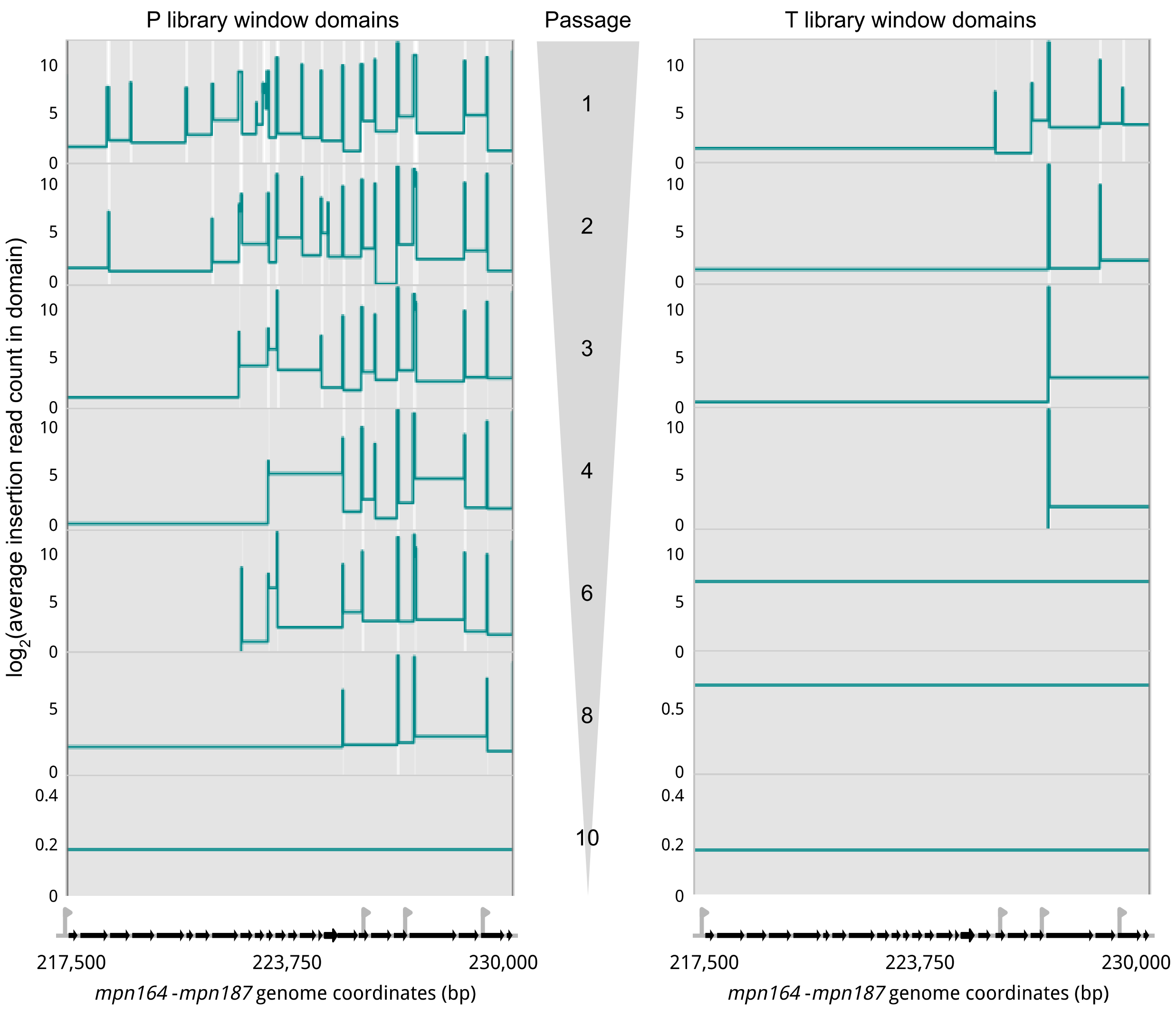

Appendix Figure S7 - Transposon insertion windows domains from passage 1 to 10 across the operon containing genes *mpn164* to *mpn187* mainly encoding ribosomal proteins. This corresponds to the same representation as panel C in Fig. 2, but showing the window essentiality domains across increasing passages (top to bottom), highlighting the differential persistence of each transposon library depending on their proximity to the operon TSS. Gray and white areas represent E and NE regions, respectively. The height of each domain corresponds to the log2 transform of the average of transposon read count per insertion.

Appendix Figure S8

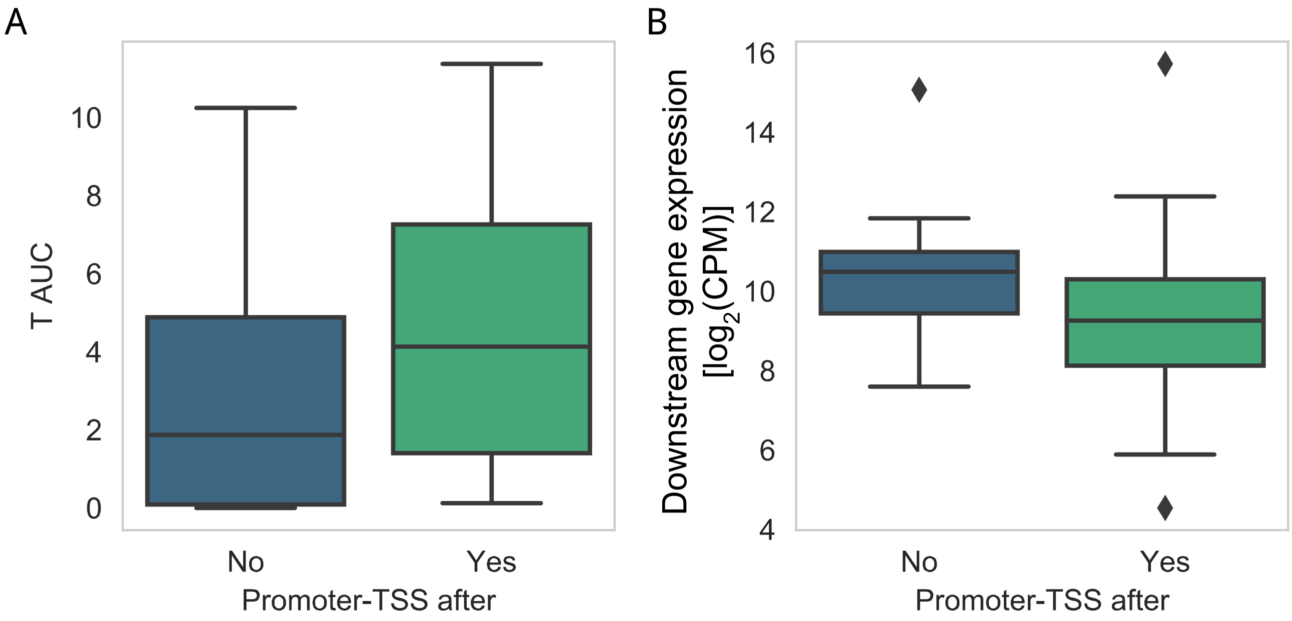

Appendix Figure S8 - Tolerance of transcription termination depending on the transcriptional context. A) Box plot comparing the persistence (AUC in Y-axis) of transposons containing terminators in iGiO elements preceding E genes and separated in the X-axis by the presence (green box) or not (blue box) of a promoter and TSS that could rescue transcription. **B)** Box plot comparing the RNA expression levels (Y-axis, as $\log_2$-transformed read counts normalized per million reads) of E genes downstream to the same iGiO elements considered in panel A and separated by the presence/absence of rescuing promoter and TSS.

Appendix Figure S9

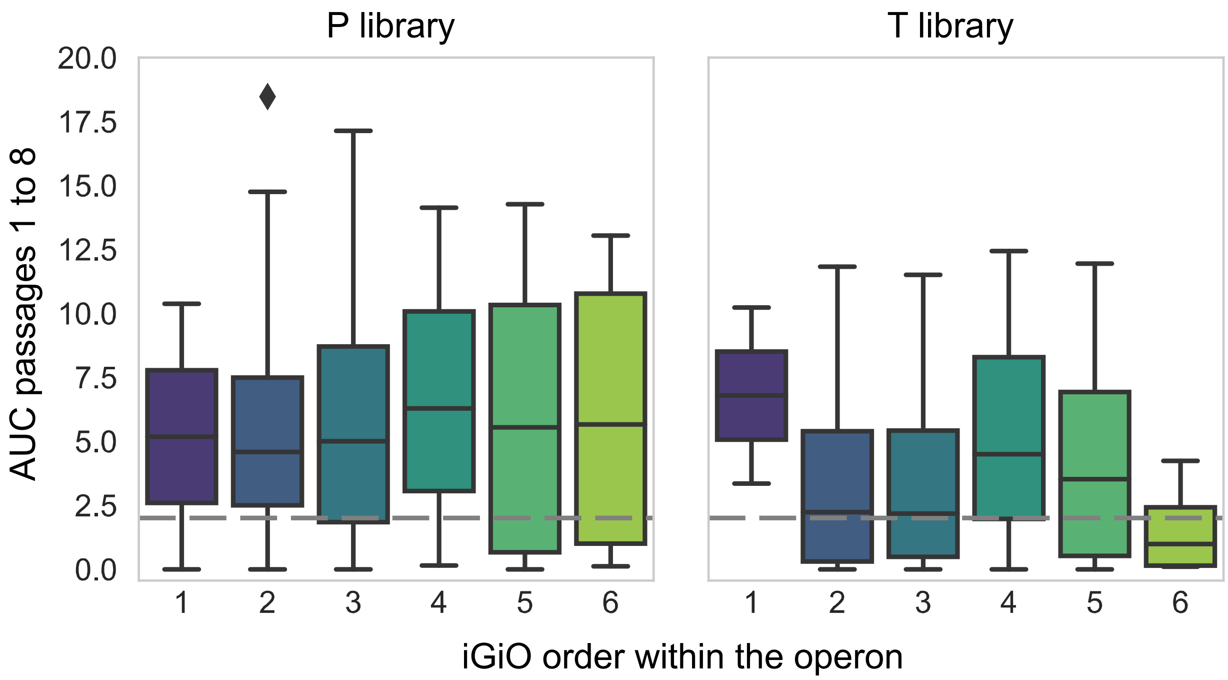

Appendix Figure S9 - Transposon insertion persistence across passages (1 to 8) for P and T libraries considering iGiO elements preceding E/F1 genes. Box plots show the persistence (AUC in Y-axis) of transposons containing promoters (left) or terminators (right) in iGiOs elements depending on their relative position (X-axis) in the transcriptional unit (*e.g.*, 3 means it is the third iGiO within a transcriptional unit).

Appendix Figure S10

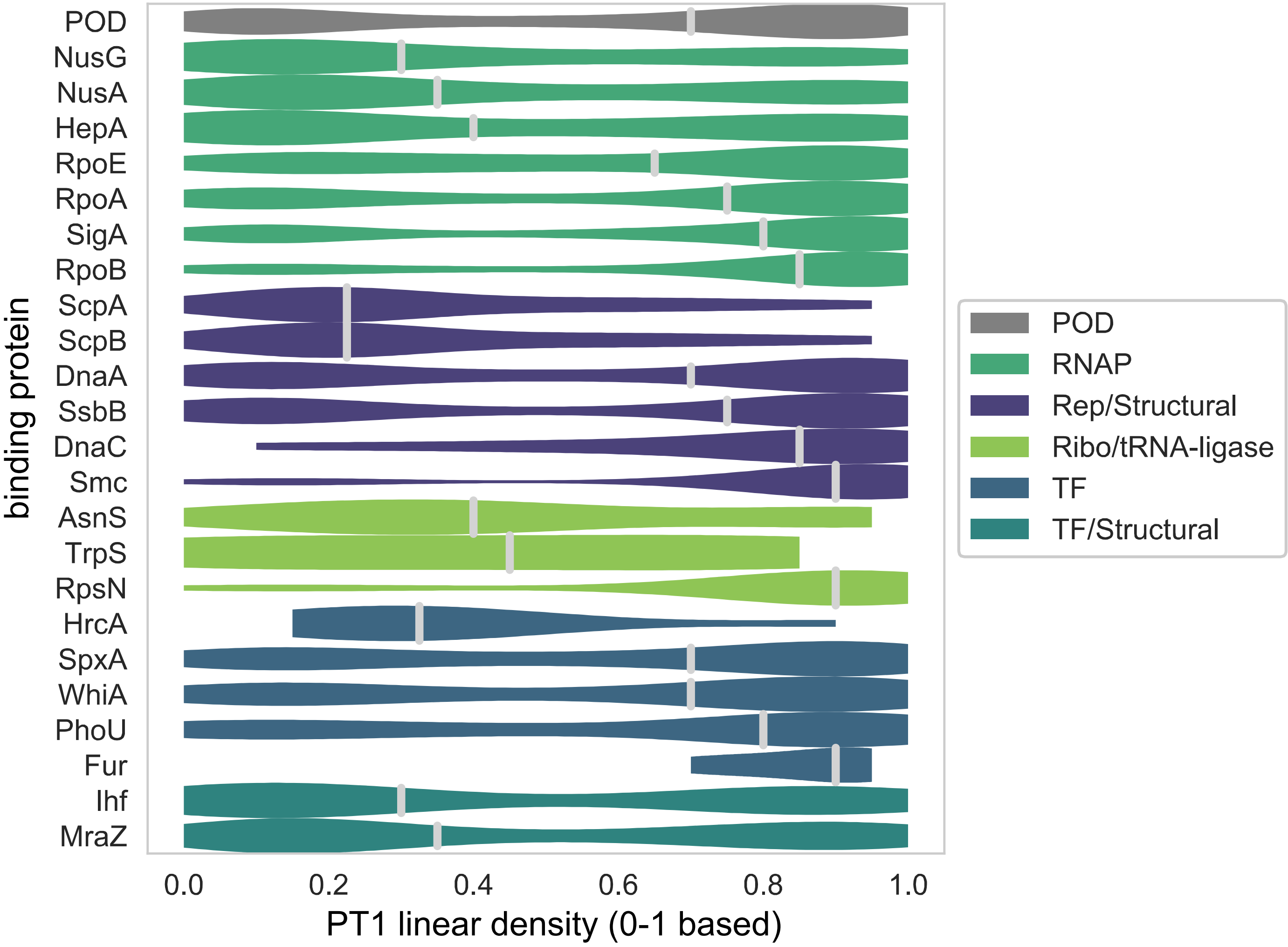

Appendix Figure S10 - Linear densities distribution at passage 1 in library PT for DNA protection (POD) data and ChIP-seq of specific DNA-binding proteins. Horizontal violin plot shows the transposon linear density distribution for different binding and structural motifs (Y-axis), colored by the type (see legend).

Appendix Figure S11

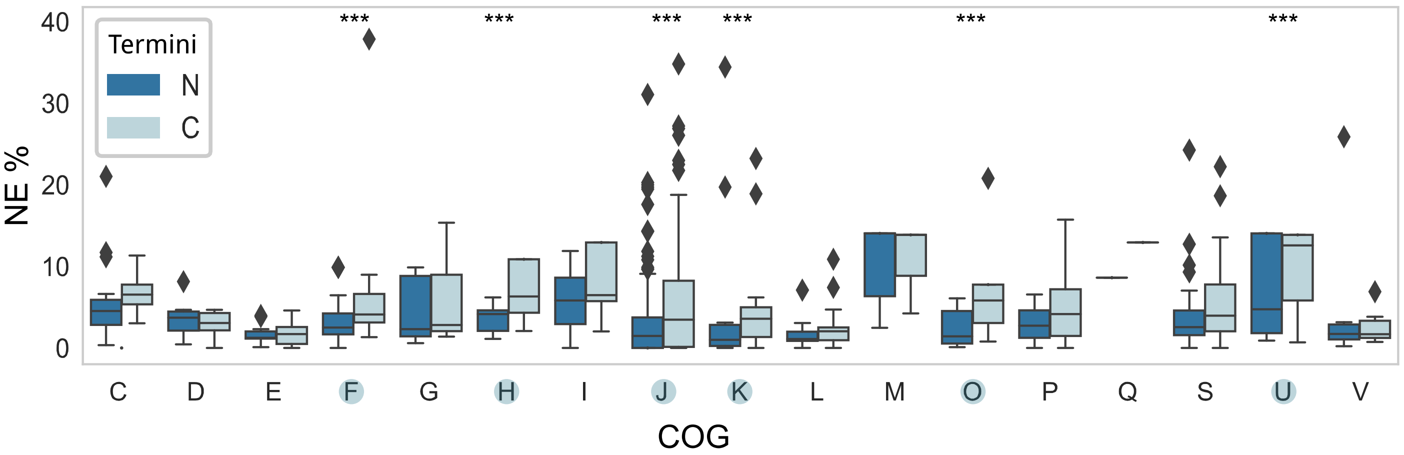

Appendix Figure S11 - COG category analysis of N- and C- terminal extensions for E and F1 genes. Box plots represent the percentages of NE segments at PT1 condition within the N- or C-terminal extensions of E and F1 genes, which are classified by COG category (X-axis). Notice that some COG categories are missing as no E/F1 are included in those groups. COG categories presenting significant differences (Wilcoxon Test, P-value < 0.05) between both termini are marked with the asterisks and in a blue circle in the X-axis labels. These are: F - Nucleotide metabolism and transport; H - Coenzyme metabolism; J - Translation; K - Transcription; O - Post-translational modification, protein turnover, chaperone functions; and U - Intracellular trafficking and secretion. Note ribosomal genes (J) present the largest values for both ends.

Appendix Figure S12

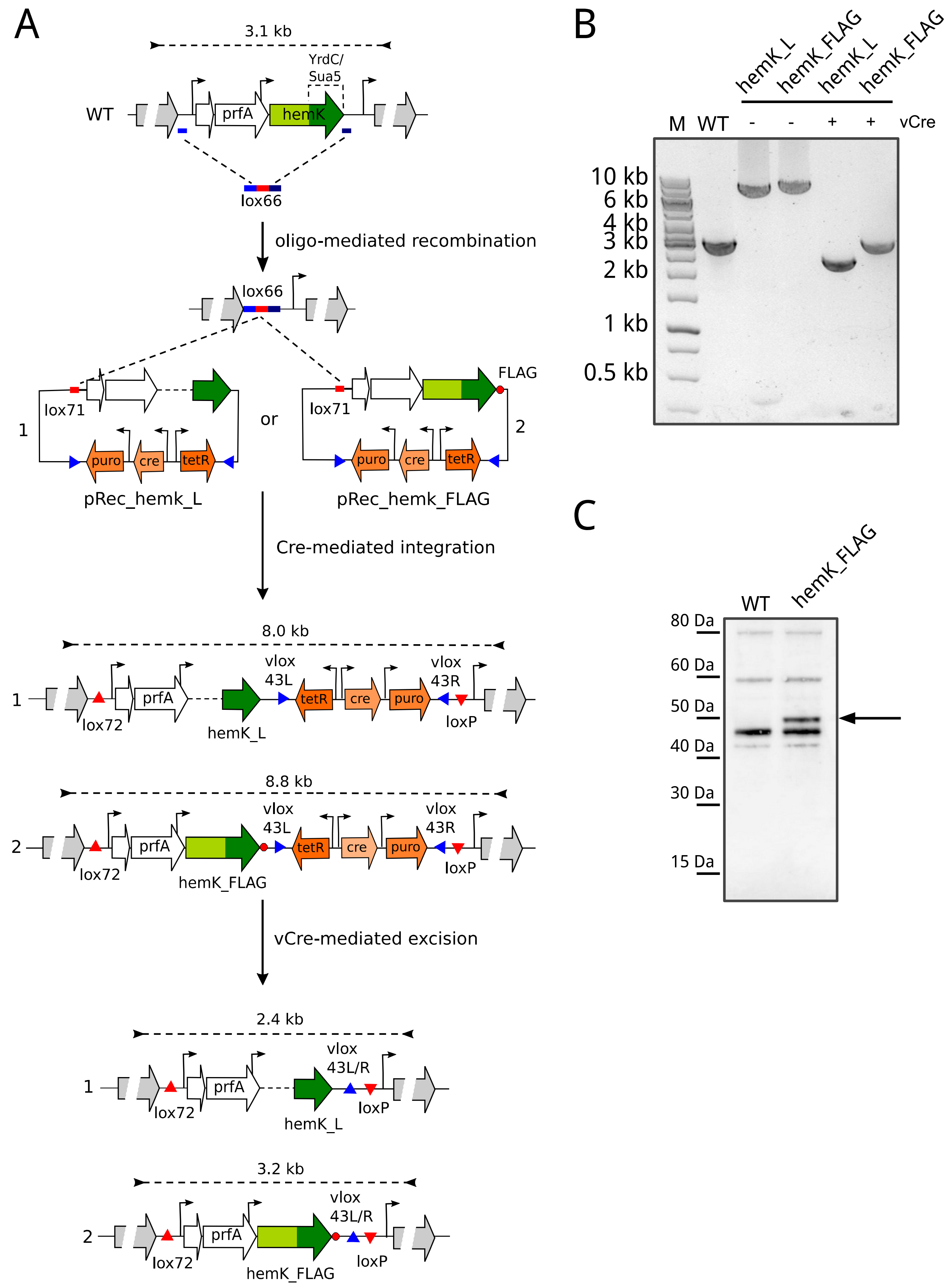

Appendix Figure S12 - Construction of *M. pneumoniae* strains expressing *mpn362* gene variants to assess the requirement of the two predicted functional domains for cell viability.

A) Schematic diagram showing the constructs and SURE-editing procedure used to generate *M. pneumoniae* strains expressing two *mpn362* gene (hemK) variants. Plasmids pRec_hemk_L and pRec_hemk_FLAG were constructed to replace the endogenous *hemK* (*mpn362*) locus by variants lacking the N-terminal Hemk domain (hemk_L), or containing a C-terminal flag (hemk_FLAG), respectively. Note that both plasmids also contain the *mpn360* (*rpmE*) and *mpn361* (*prfA)* genes to maintain the same genetic architecture after complementation. Briefly, deletion of the *rpmE-prfA-hemK* (*mpn360-mpn361-mpn362*) endogenous locus is mediated by oligo-recombineering using an oligo containing a lox66 recombination site (in red) flanked by homologous regions (in blue). Gene complementation with the mutant variants is then mediated by Cre-mediated integration of the plasmids mentioned above in the lox66 site, generating lox72 and loxP sites. Plasmid backbone (containing tetR repressor, Cre and puromycin resistance marker) is then removed from the genome by vCre-mediated recombination using the vlox sites present in the plasmid sequence. Sizes of the PCR products expected for each intermediate strain are shown above.

B) PCR analyses using genomic DNA of the WT and intermediate strains before (labeled as "-") and after (labeled as "+") vCre excision are shown. The size of the expected PCR products is shown in panel A

C) Western blot analysis of cell lysates of the WT and hemk_FLAG mutant using anti-FLAG antibodies. The arrow shows the protein band expected for the expression of the full-length *mpn362* coding-sequence, indicating that it is expressed as a fusion protein.

Appendix Figure S13

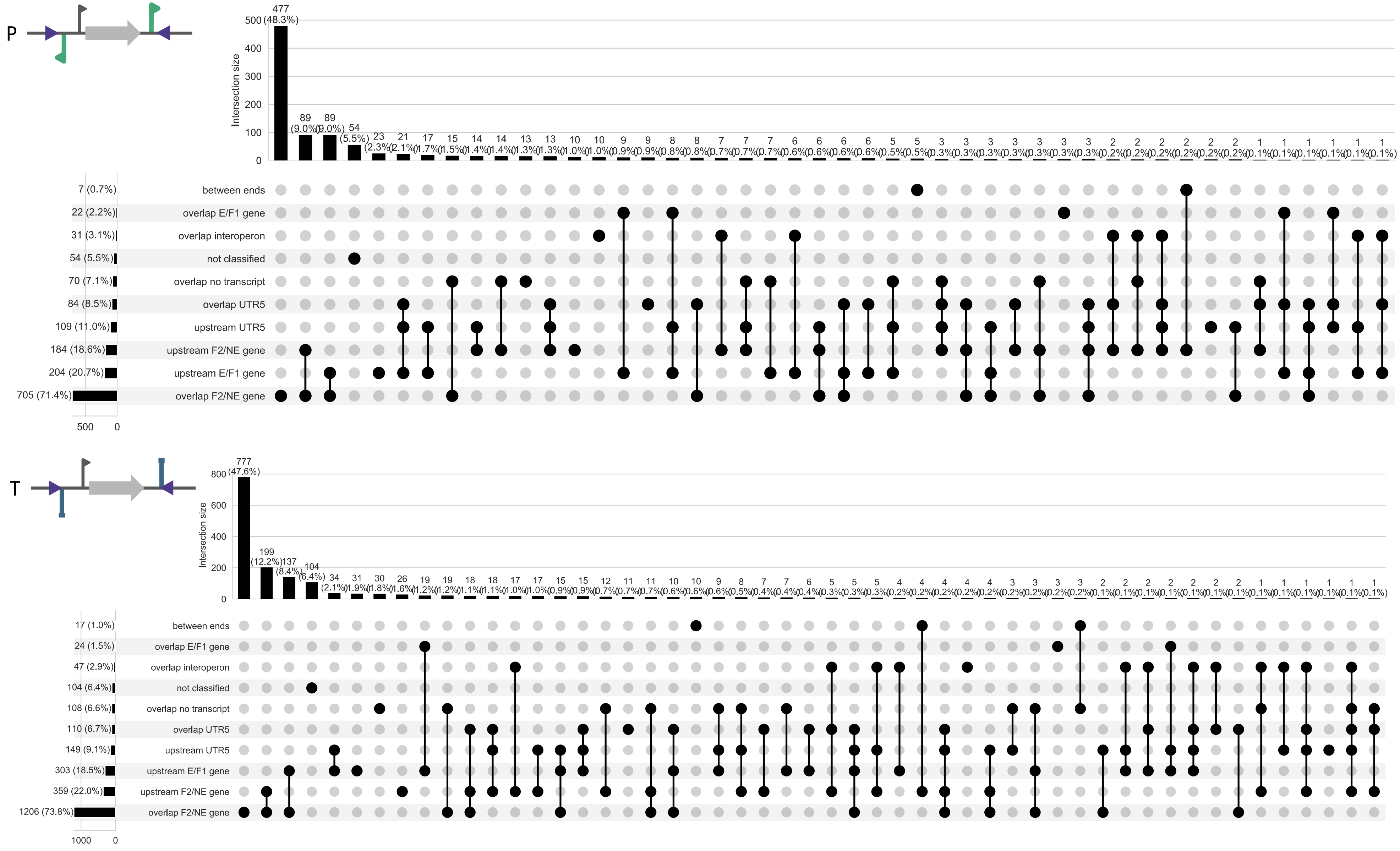

Appendix Figure S13 - Upset plots representing the different contexts where unique P or T insertions are maintained at nucleobase level. P- and T- selected positions are represented in the top and bottom panels, respectively (associated to construct schemes). Left bar plots account for the different contexts where these insertions are found, represented as total number and percentage (between parentheses). Then, an intersection graph is presented to capture all the possible scenarios with a black solid circle linked with lines when present in several contexts. Top bar plots on each figure account for the number of events in each set.

Appendix Figure S14

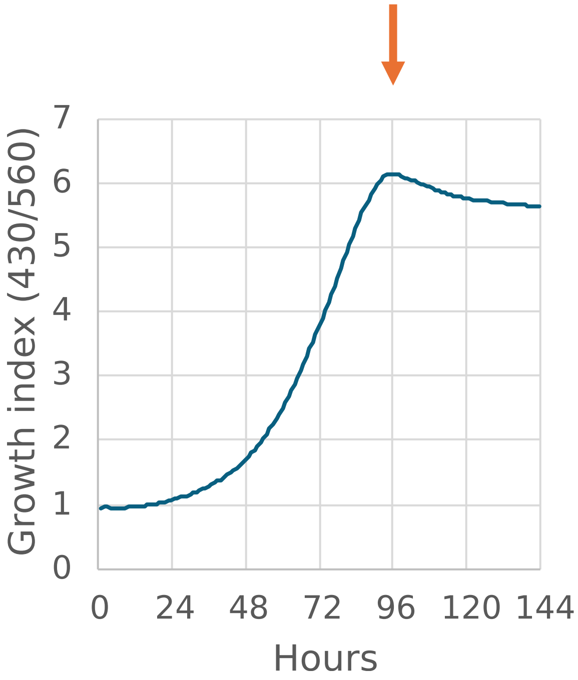

Appendix Figure S14 - Representative growth curve analysis during a single passage. The arrows indicate the time of harvesting for the next passage.

Appendix Figure S15

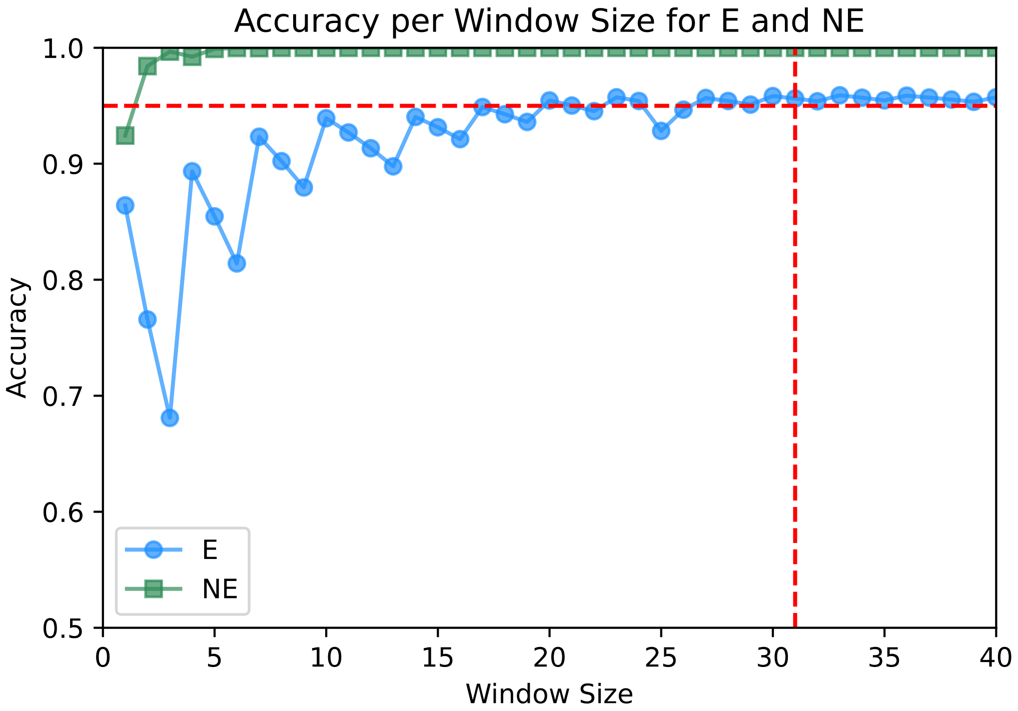

Appendix Figure S15 - Window size selection analysis. The plot displays the accuracy, calculated as the total number of right essentiality calls / total number of windows, in assigning the label E (blue) or NE (green) to windows derived from the gold set of genes with known essentiality in *M. pneumoniae*. Red dashed lines show the 0.95 accuracy and the selected size, as horizontal and vertical annotations, respectively.
