# Supplementary material for: Quantitative essentiality in a reduced genome: a functional, regulatory and structural fitness map
Source: Mol Syst Biol. 2025 Aug 13;21(10):1388–416. doi: 10.1038/s44320-025-00133-1 (PMC12494982; doi:10.1038/s44320-025-00133-1)
Supplement: Supplementary file 21 — Expanded View Figures [file 44320_2025_133_MOESM21_ESM.pdf]

Expanded View Figures

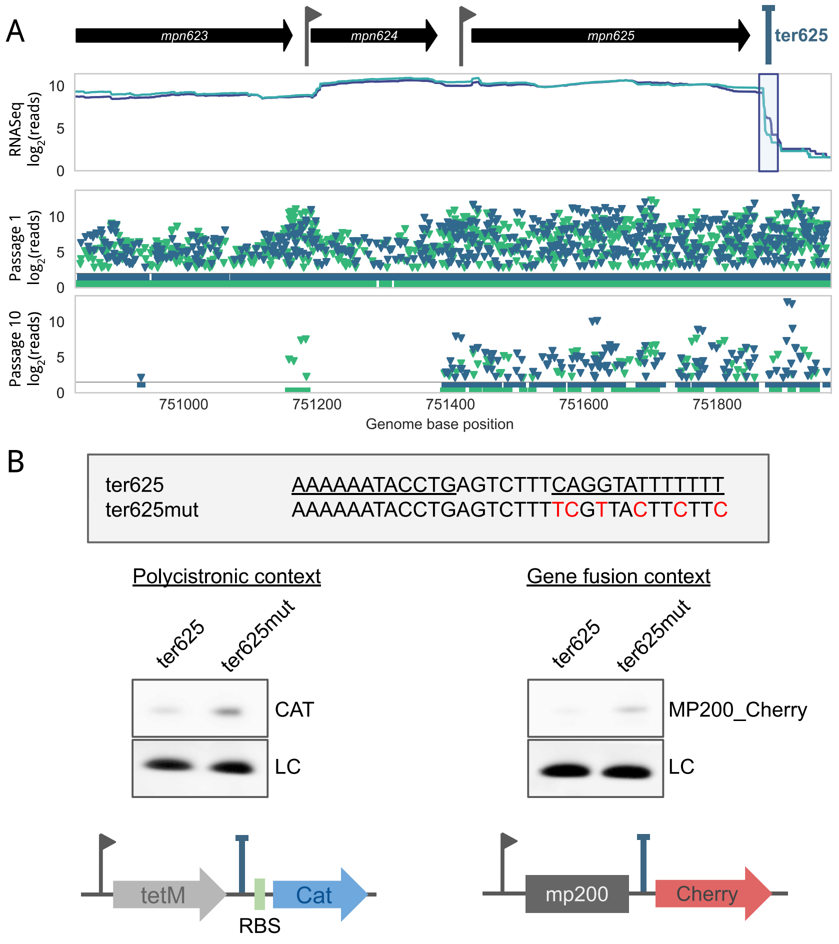

Figure EV1. Genomic context and activity of the endogenous ter625 terminator.

(**A**) Scheme showing the position of the ter625 terminator sequence (downstream blue box, with a blue vertical line representing the start of the hairpin) in its natural genomic context. Genes appear as black arrows, and gray flags indicate the position of predicted promoters and TSS. Below is shown the RNAseq expression profile ($n = 2$), and transposon insertion mapping at passages 1 and 10 are shown along the genomic region. Green and blue triangles represent transposon insertions containing promoters or terminators, respectively. Note the enrichment of transposons containing terminators after ten passages next to the position of the endogenous ter625 terminator. (**B**) Termination activity of the ter625 sequence in two different genetic contexts using gene reporters expressed as polycistronic or fusion transcripts. On top of the panel is shown the wild-type ter625 sequence compared to a mutated version affecting the terminator hairpin structure (underlined). The expression of *cat* or cherry gene reporters was assessed by Western blot analysis in the two genetic configurations shown below the panel. The gray flags indicate the promoter of the transcriptional unit, while the position of the ter625 sequence is shown as a blue T, just before a ribosome binding site (RBS, left panel) or after the mp200 polypeptide fusion (right panel).

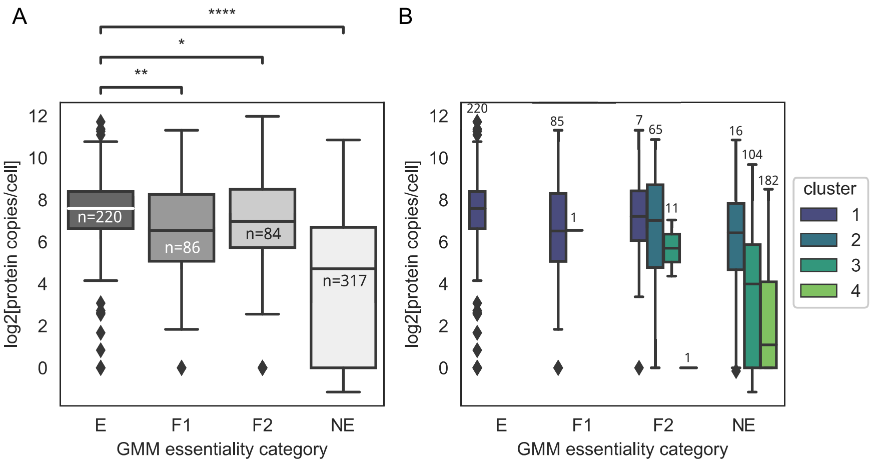

Figure EV2. Box plots showing the relation between GMM class and k-means clusters in relation to protein copy numbers.

(**A**) For each predicted essentiality category at PT1 (X-axis), we relate the $\log_2$-transform copies per cell of the proteins in each group (Y-axis). E genes present significantly higher copies/cell levels than F1, F2, and NE genes ('*' Mann–Whitney $P < 0.05$; '****' Mann–Whitney $P < 0.0001$). NE genes present the lowest copies per cell. Box plots show the median (center line), the 25th and 75th percentiles (box bounds), and the minimum and maximum values (whiskers), or display outliers as diamonds, following the default settings of the Seaborn's boxplot function. The sample size of each category is labeled within the box. (**B**) Same representation as panel (**A**) but separating genes by the cluster assigned by the k-means applied on the LD decays between PT1 and PT8. It can be observed that F2 and NE genes assigned to higher clusters (*i.e.*, more stable insertions) present lower copies per cell. Box plots show the median (center line), the 25th and 75th percentiles (box bounds), and the minimum and maximum values (whiskers), or display outliers as diamonds, following the default settings of the Seaborn's boxplot function. On top of each box, the sample size is labeled. Note that for the clustering analysis, we excluded 15 NE genes with a high percentage of sequence repetition, as these interfere with the clustering prediction (see Methods). In addition, we assigned a value of 0.0 copies/cell to proteins not detected (labeled as "-" in Dataset EV2).

 © The Author(s)

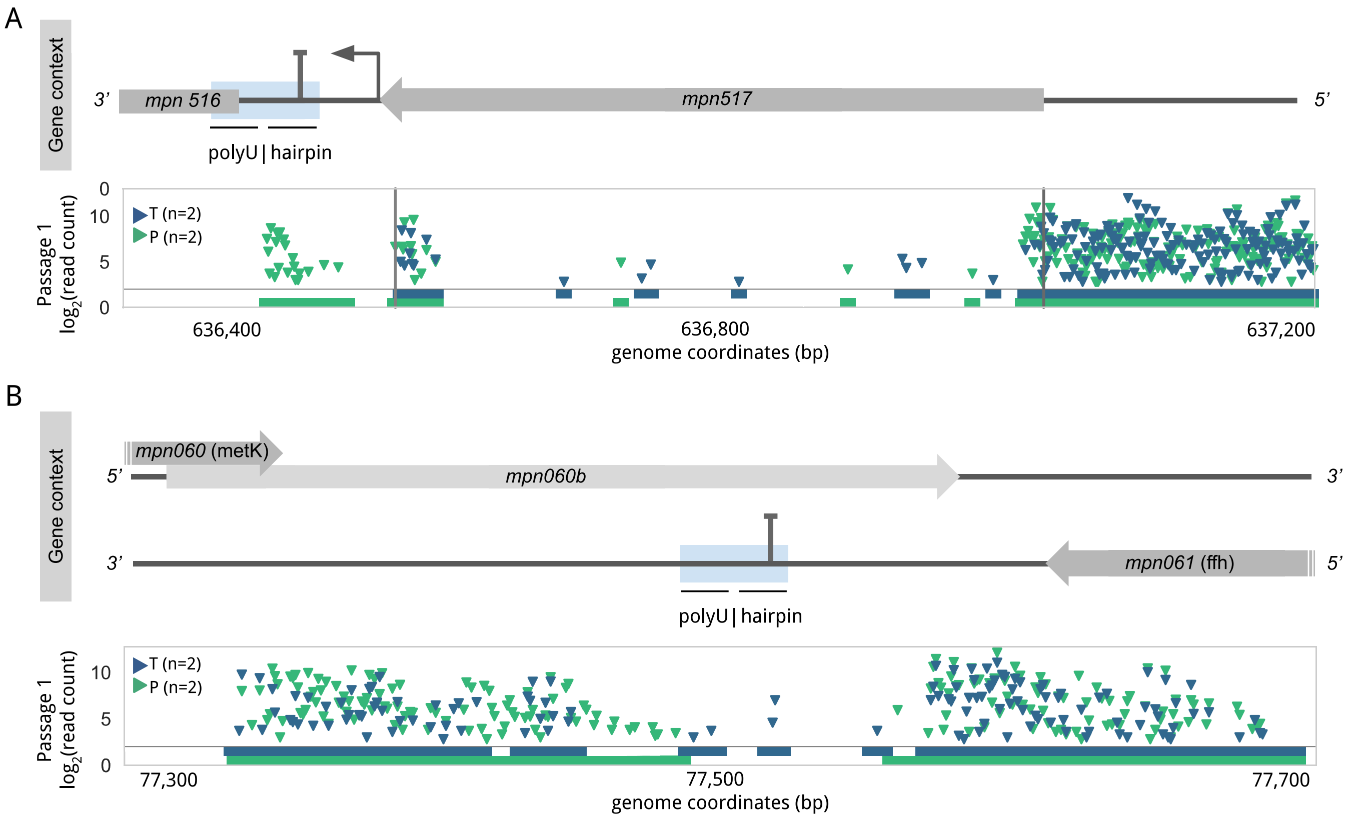

Figure EV3. Examples of essential 3′UTR and terminator signals.

Representation of the insertion profiles and genomic context of (**A**) *mpn517* and (**B**) *mpn061*, two genes presenting essential 3′UTRs. For each panel, the insertion profile of the region of interest is shown. Transposons containing promoters or terminators are depicted in blue and green triangles, respectively. The genetic context is also shown on top of each panel, including the orientation of the genetic elements. Predicted intrinsic terminator sequences are labeled in blue boxes, showing hairpin and poly-U sequences. The 3′UTR are defined from stop codons to the terminators. It should be noticed that the 3′UTR region of *mpn517* partially overlaps with the 5′UTR of *mpn516*, which is essential in the T library but not in the P library, thus making it difficult to assess whether it is essential the terminator or the 5′UTR.

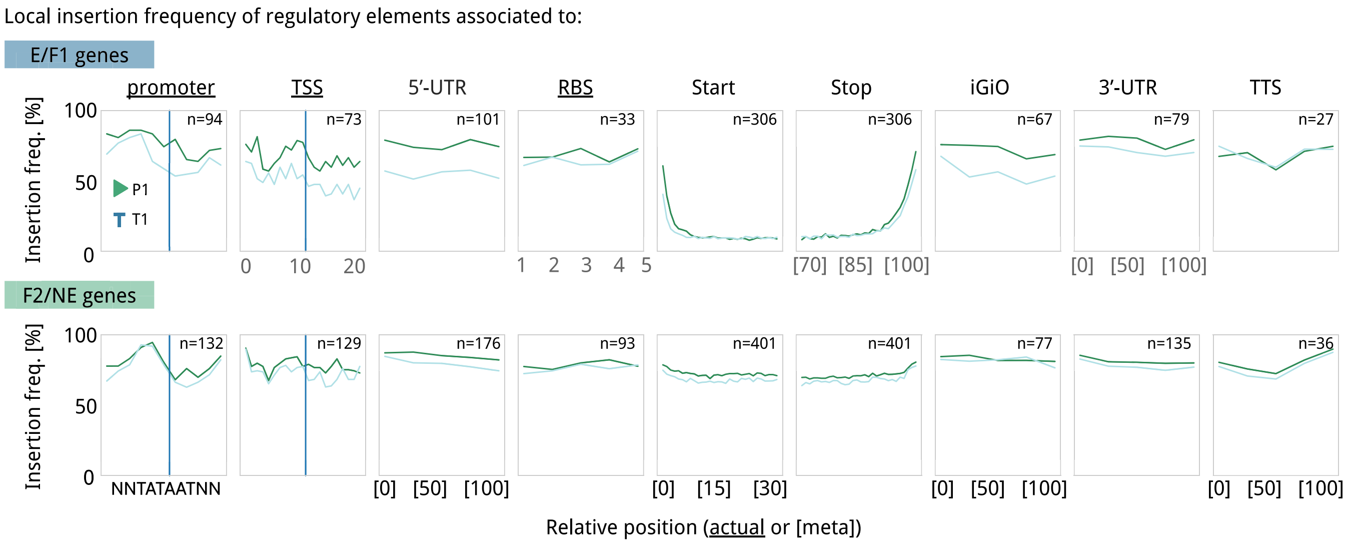

Figure EV4. Local transposon insertion frequency in different regulatory elements.

The local frequency of transposon insertions containing promoters (green) or terminators (blue) at passage 1 is shown for different regulatory elements (including start and stop codons) associated with E/F1 (top panel) or F2/NE genes (bottom panel). Regulatory elements with a fixed sequence length are underlined and have a solid blue line representing the center of the element. For regulatory elements with a variable length, the X-axis represents the relative percentage of the covered region (with a minimum of 5 bins for regulatory elements and 100 bins for genes, and then we represent the first 30 and last 30 bins to get the start and stop regions). While no difference is observed for F2/NE genes, E and F1 genes are characterized by an enrichment of transposons containing promoters in upstream regulatory elements, except for RBS. Downstream regulatory elements (3′-UTR and TTS) do not show significant global differences regardless of the essentiality of the associated gene. Note that the N- and C-terminal regions of E genes differ in the length of transposon disruption tolerance, with C-terminal regions showing a higher tolerance for extended regions.

© The Author(s)

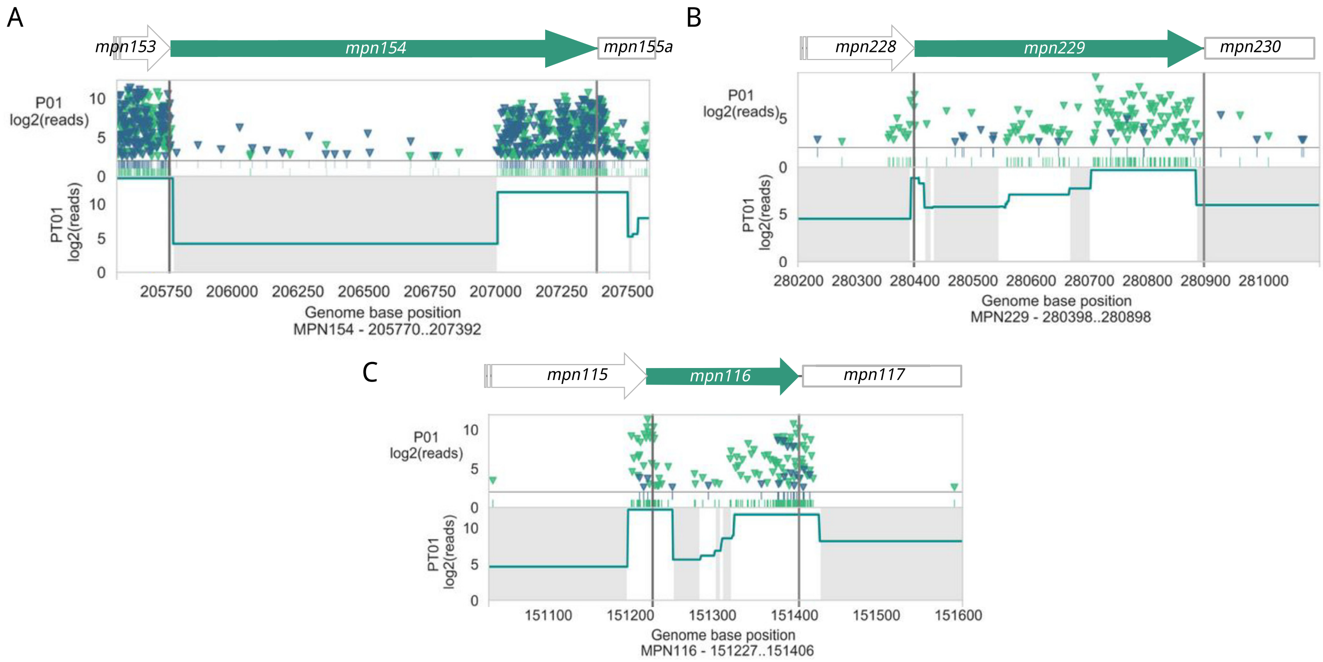

Figure EV5. Examples of extended termini regions in E genes.

Panels correspond to genes *mpn154* (**A**), *mpn229* (**B**), and *mpn116* (**C**). For each panel, the insertion profile of the region of interest at passage 1 is shown. Transposons containing promoters or terminators are depicted in blue and green triangles, respectively. Vertical lines show start and stop codons of the gene, also including their genomic context on top.
